# Chimeric antigen receptor T cells for gamma–delta T cell malignancies

**DOI:** 10.1038/s41375-021-01385-0

**Published:** 2021-08-13

**Authors:** P. A. Wawrzyniecka, L. Ibrahim, G. Gritti, M. A. Pule, P. M. Maciocia

**Affiliations:** 1 Department of Haematology, Cancer Institute, University College London, London, UK; 2 Hematology Department, Azienda Socio Sanitario Territoriale Papa Giovanni XXIII Hospital, Bergamo, Italy

**Keywords:** T-cell lymphoma, Acute lymphocytic leukaemia

## To the Editor:

Cancers derived from the malignant transformation of gamma–delta (γδ) T cells carry very poor prognosis. The major pathologies recognised are γδ T acute lymphoblastic leukaemia (γδ T-ALL), and two lymphoma subtypes: hepatosplenic T cell lymphoma (HSTL) and primary cutaneous γδ T cell lymphoma (PCγδ-TCL) [1]. γδ T-ALL represents approximately 10% of cases of T-ALL and is associated with high rates of induction failure, relapse and excess mortality [2]. HSTL is a rare (approximately 3% of cases of T cell lymphoma [1]) but highly aggressive disorder, which typically presents in males in the 2nd or 3rd decade of life, often in association with immunosuppressive therapy [3]. It carries the worst prognosis of all lymphoma subtypes, with a median survival of only 6–8 months [4] and only isolated cases of long-term survival [5]. PCγδ-TCL is also rare (approximately 1% of skin lymphomas [1]) and presents with cutaneous involvement, typically associated with visceral and/or bone marrow disease. Again, outcomes are poor, with 75% 1-year mortality in the largest published case series [6].

Treatment for γδ malignancies is with cytotoxic chemotherapy, with no tumour-specific therapies currently available. By contrast, in analogous B-cell malignancies, highly effective immunotherapies, including monoclonal antibodies, bispecific T cell engagers and chimeric antigen receptor (CAR)-T cells [7] are available. These therapies have revolutionised the treatment and outcome of advanced B-cell malignancies. CAR-T cells against CD19 in particular have demonstrated the potential to induce long-lasting complete remissions even in patients with advanced and refractory cancers [7].

For γδ malignancies, the defining immunophenotypic characteristic is expression of the γδ T cell receptor (TCR), present in >95% of cases of HSTL and in all PCγδ-TCL and γδ T-ALL [3]. Importantly, in normal tissues expression is limited to γδ T cells, where it functions as the antigen recognition receptor. Here, we developed CAR-T cells targeting the γδ TCR and demonstrate in vitro and in vivo efficacy against γδ T cell malignancies. Our data offers proof-of-concept for γδ TCR-targeting with CAR-T cells as a potential approach to bring highly potent immunotherapy to the treatment of γδ malignancies.

**Fig. 1:**
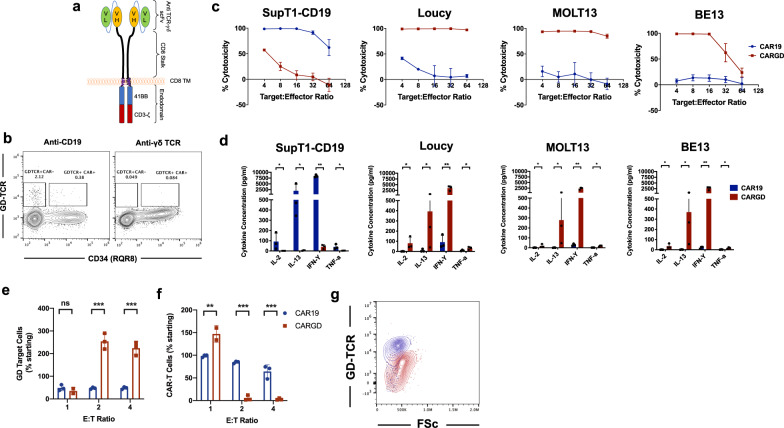
In vitro testing of anti-γδ TCR CAR. **a** Schematic of anti-γδ TCR CAR, with 2nd generation architecture. **b** Example flow plot of γδ-TCR staining on anti-CD19 or anti-γδ TCR CAR-T cells following transduction **c** co-cultures of anti-γδ TCR or control anti-CD19 CAR-T cells with CD19+ (SupT1-CD19) or γδ TCR+ cell lines (Loucy, MOLT13, BE13) (**c**) cytotoxicity at 72 h, as measured by bioluminescence-based assay (**d**) cytokine secretion at 48 h. **e**–**g** 120-h co-culture of control or anti-γδ TCR CAR-T cells with autologous normal γδ T cells, *n* = 3. **e** Residual γδ T cells as proportion of starting cells **f** Example γδ TCR staining on normal γδ T cells after co-culture with anti-γδ TCR CAR-T or anti-CD19 CAR-T cells **g** residual anti-γδ TCR CAR-T or anti-CD19 CAR-T following co-culture, as proportion of starting cells. **p < 0.001, ***p < 0.0001.

Primary αβ T cells were retrovirally transduced to express anti-γδ TCR CAR or control anti-CD19 CAR (Fig. 1a). Following transduction with anti-CD19 CAR, a small proportion of γδ T cells persisted in the culture, including some which expressed anti-CD19 CAR. By contrast, for anti-γδ TCR CAR, no γδ T cells were detected in the culture, suggesting ‘purging’ of these cells by the transduced population (Fig. 1b). CAR-T cells were then co-cultured with T cell lines which natively express (Loucy – Vγ9Vδ2, BE13 – Vγ8Vδ1, MOLT13 – Vγ3Vδ1 [8]) or are negative for surface γδ TCR (SupT1-CD19). While control anti-CD19 CAR lysed only SupT1-CD19 cells, anti-γδ TCR CAR-T lysed only γδ TCR-positive cell lines (Fig. 1c). In addition, anti-γδ TCR CAR-T cells demonstrated specific secretion of cytokines including interferon-γ, IL-2, IL-13 and TNF-α (Fig. 1d). Next, we co-cultured anti-CD19 or anti-γδ CAR-T cells with normal autologous γδ T cells. At a high E:T ratio (1:1), target normal γδ T cells were partially lysed (Fig. 1e), with concomitant expansion of anti-γδ CAR-T cells (Fig. 1f). A marked downregulation of γδ TCR expression was noted on surviving γδ T cells (Fig. 1g). Interestingly, by contrast, at lower E:T ratios (1:2 and 1:4), paradoxical γδ T cell expansion was instead observed (Fig. 1e), associated with reduction in numbers of anti-γδ CAR-T cells (Fig. 1f). This suggests lysis of anti-γδ CAR-T by target normal γδ T cells.

**Fig. 2:**
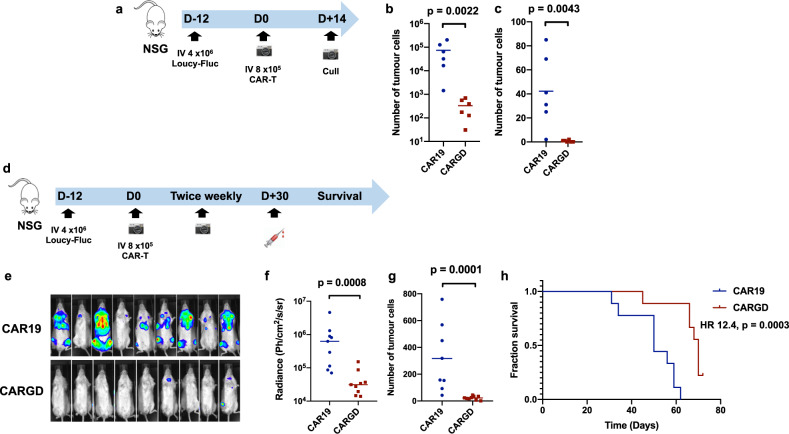
In vivo assessment of anti-γδ TCR CAR. **a** Schematic of Loucy murine model (*n* = 6/group) **b** Quantification of tumour in (**b**) marrow and (**c**) spleen at D14 following CAR-T injection. **d** Schematic of Loucy murine model (n = 9/group) **e** bioluminescence (BLI) imaging at D21 following CAR-T infusion. **f** Quantification of BLI signal at D21 **g** quantification of tumour burden in blood at D30 **h** survival curves of mice (median survival CAR19 50 days v CARγδ 69 days, HR 12.4, *p* = 0.0003, comparison by log-rank method). All other comparisons by Mann–Whitney *U* test.

To assay the in vivo potency of anti-γδ TCR CAR-T cells, we utilised the Loucy murine model of disseminated γδ TCR-positive leukaemia (Fig. 2a, d). NSG mice were intravenously injected on CAR D-12 with 4 × 10^6^ Loucy cells, engineered to stably express Firefly luciferase. Tumour engraftment was confirmed by bioluminescence imaging (BLI) at D-1 (data not shown), then mice were treated on D0 with 8 × 10^5^ anti-γδ TCR or control anti-CD19 CAR-T cells. Mice receiving anti-γδ TCR CAR demonstrated reduction of tumour burden, as assessed by flow cytometry of bone marrow and spleen at necropsy on D14 (Fig. 2b, c, Supplementary Fig. 1), BLI (Fig. 2e, f) and bleed at D30 (Fig. 2g). Prolonged survival (Fig. 2h) was seen in anti-γδ TCR CAR recipients compared to CD19 CAR-treated animals, although all animals eventually died of progressive γδ TCR-positive disease, with no evidence of antigen downregulation. In common with other NSG models, CAR-T cell persistence was limited, with no detectable cells in the peripheral blood at D30 (data not shown).

Despite success in B-cell malignancies, a lack of acceptable targets means targeted immunotherapy is rarely applied to T cell malignancies. The anti-CD30 antibody-drug conjugate brentuximab vedotin is effective in anaplastic large cell lymphoma [9], however, CD30 is not typically expressed in γδ cancers [2]. Suggested approaches to targeting T cell malignancies include targeting pan-T cell antigens such as CD5 [10] or CD7 [11]. However, such strategies deplete the entire normal T cell compartment and may induce profound immunosuppression [11], potentially requiring rescue by allogeneic hematopoietic stem cell transplant.

More refined approaches that target clonal elements of the TCR, such as selective targeting of TRBC1 and TRBC2 in αβ T cell malignancies, allow depletion of only part of the normal T cell compartment [12]. While analogous approaches are potentially possible in γδ TCR malignancies, simple targeting of the γδ TCR may be feasible. This approach could concomitantly deplete normal γδ T cells. These constitute <5% of peripheral blood T cells, are more abundant in tissues and have a range of proposed ancillary immunological functions [13]. Importantly, genetically γδ-deficient mice display a very mild phenotype [14], and there is no known human pathology associated with γδ T cell deficiency. This suggests that depletion of the γδ T cell compartment may be clinically tolerable, although initially clinical exploration of anti-γδ TCR CAR-T should proceed cautiously: for instance with co-expression of a suicide gene [15], availability of back-up cryopreserved peripheral blood mononuclear cells, and close monitoring for development of atypical infections.

Indeed, it is unclear if anti-γδ CAR-T treatment would lead to γδ T cell aplasia. An interesting observation in our study was that, while anti-γδ CAR-T expanded when cultured with normal γδ T cells at high E:T ratio, the reverse was observed when normal γδ T was in excess. Thus, anti-γδ CAR-T were themselves depleted from the culture, with expansion of normal γδ T cells. The probable explanation is that CAR binding to the TCR of γδ T cells induced CAR signalling, but also signalling via the TCR of the γδ T cell, leading to a 2-way synapse with each cell potentially both target and effector. When normal γδ T outnumbered anti-γδ CAR-T, the balance of cytotoxicity resulted in anti-γδ CAR-T cell lysis and expansion of the normal cells. The potential clinical consequences for anti-γδ TCR CAR therapy are unclear and would be difficult to ascertain pre-clinically due to a lack of relevant immunocompetent models. However, in patients receiving anti-γδ CAR-T, it is likely that the CAR-T: normal γδ T cell ratio at the tumour site would be high in the critical initial CAR-T expansion phase, following lymphodepleting chemotherapy.

Here, we have demonstrated the feasibility of engineering normal αβ T cells to express anti-γδ TCR CAR and have shown that anti-γδ TCR CAR-T cells can specifically kill malignant γδ cells both in vitro and in vivo. Our approach offers the first proposed strategy to bring highly potent CAR-T cells to the treatment of γδ T cell malignancies, where there is a major unmet need for effective therapies. Clinical assessment of this approach is warranted.

## Supplementary information

Supplementary Methods/ Results

Supplementary Figure 1
