# Supplementary material for: Chimeric antigen receptor T cells for gamma–delta T cell malignancies
Source: Leukemia. 2021 Aug 13;36(2):577–9. doi: 10.1038/s41375-021-01385-0 (PMC8807386; doi:10.1038/s41375-021-01385-0)
Supplement: Supplementary file 1 — Supplementary Methods/ Results [file 41375_2021_1385_MOESM1_ESM.docx]

**Supplementary Methods/ Results**

*Specificity testing of anti-γδ TCR scFv*

The murine anti-γδ TCR G5-4 scFv recognises a common epitope present on all γδ T cells (Wei *et al*, China Patent CN103130894A, 2011). Specificity of the scFv for γδ T cells, and no other peripheral blood mononuclear cells (PBMCs), was confirmed by cloning of the scFv as a fusion protein with a murine IgG2a-Fc tag. HEK-293T cells were transiently transfected to produce soluble scFv-Fc protein and culture supernatant containing the fusion protein was incubated with PBMCs from normal donors. After staining with anti-Fc secondary, binding was seen only on <5% of CD4-CD8-CD3+ T cells, corresponding to the proportion of γδ T cells separately identified in the same donors using a commercial antibody. The G5-4 scFv is not suitable for use in formalin-fixed paraffin embedded (FFPE) tissues and so a formal tissue cross-reactivity study could not be performed.

*Construction of anti-*γδ *TCR and control CAR T-cells*

The murine anti-γδ TCR scFv G5-4 was cloned into a 2^nd^ generation (CD8-stalk and transmembrane domain/ 41BB-TCRzeta endodomain) CAR architecture, in SFG plasmid with the RQR8 sort-suicide gene^1^. RQR8 incorporates epitopes from human CD34 and CD20, and is recognised by the anti-CD34 antibody clone qBend10, and the monoclonal antibody rituximab^8^. The G5-4 scFv recognises a common epitope present on all γδ T cells (Wei *et al*, China Patent CN103130894A, 2011). The control anti-CD19 CAR employed an identical architecture but with the FMC63 anti-CD19 scFv, as utilised in tisagenlecleucel and axicabtagene ciloleucel.

*Cell lines*

HEK-293T were cultured in Iscove’s modified Dulbecco’s medium (IMDM) (Lonza, Basel, Switzerland) supplemented with 10% FBS (HyClone, GE, Buckinghamshire, UK) and 2 mM GlutaMAX (Invitrogen, CA). SupT1, SupT1-CD19, BE13, MOLT13 and Loucy cell lines were cultured in complete RPMI (RPMI-1640, Lonza, Basel, Switzerland) supplemented with 10% FBS and 2 mM GlutaMAX). Cells were maintained in a humidified atmosphere containing 5% CO2 at 37 °C. All cell lines were obtained from the American Type Culture Collection (ATCC), Deutsche Sammlung von Mikroorganismen und Zellkulturen (DSMZ) or Public Heath England (PHE) collections. Luciferised SupT1, BE13, MOLT13 and Loucy cell lines were produced by retroviral transduction with a plasmid expressing HA marker gene and Firefly luciferase (FLuc). SupT1-CD19 were produced from parental SupT1 by retroviral transduction with a plasmid expressing C-terminal truncated CD19, HA marker gene and Fluc, followed by single cell cloning of positive clones. All cell lines were routinely tested for mycoplasma and for cell-surface expression of target antigens.

*Retrovirus production and transduction of primary T cells*

RD114-pseudotyped supernatant was generated as follows. HEK-293T cells were transfected with vector plasmid; RDF, an expression plasmid to supply RD114 envelope (a gift from M. Collins, University College London); and PeqPam-env, a Gag-Pol expression plasmid (a gift from E. Vanin, Baylor College of Medicine). Transfection was facilitated using GeneJuice (Merck, Darmstadt, Germany). Peripheral blood mononuclear cell (PBMC) transductions were performed as follows. PBMCs from normal donors were isolated by Ficoll (GE, Buckinghamshire, UK) gradient centrifugation and T cells were stimulated with CD3/28 antibodies (Immunocult, StemCell Technologies, Vancouver, Canada). IL-2 (GenScript, Nanjing, China; 100 IU/ml) was added following overnight stimulation. On day 3, T cells were collected, plated on retronectin (Takara, Nojihigashi, Japan) with retroviral supernatant, and centrifuged at 1,000g for 40 min. Transduction efficiency was determined on days 6–7 following initial collection, and further experiments were commenced on days 7–10 following initial collection. Transduction was determined by expression of RQR8 marker gene (qBend10 staining) or by direct detection of murine scFv using anti-murine Fab antibody. Transduced T cells were maintained in complete RPMI.

*Antibodies and flow cytometry*

Flow cytometry was performed using a BD LSR Fortessa instrument (BD Biosciences, NJ). Staining steps were performed at room temperature for 20 min with phosphate buffered saline (PBS) washes between steps. The following antibodies were used (all anti-human unless otherwise specified; clone IDs are given in parentheses): CD2 (TS1/8), CD3 (UCHT1), CD4 (OKT4), CD7 (CD7-6B7), CD8 (SK1), CD19 (HIB19), CD45 (HI30), αβ TCR (T10B9), γδ TCR (B1), murine CD45 (30-F11), HA epitope tag (18B12) all from BioLegend (San Diego, CA); CD34 (Qbend10, R&D Systems, Oxford, UK). Polyclonal goat anti-murine Fab was obtained from Jackson Laboratories. All antibodies were validated by the manufacturer for diagnostic use. At least 5,000 target events were acquired per sample. Analyses were conducted using FlowJo v10 (BD Biosciences, NJ, USA).

*Isolation and purification of primary* γδ T cells

γδ T cells were isolated from PBMCs using an anti-TCR γδ MicroBead kit (MACS; Miltenyi, Auburn, CA, USA), then eluted in complete medium containing 1 μg/mL Concanavalin A (Con A; Sigma-Aldrich, Oakville, Canada), with 10 ng/mL each recombinant human interleukin (IL)-2 (Genscript, Nanjing, China) and IL-4 (R&D Systems, Minneapolis, MN, USA). Cells were replated in complete media containing without Concanavalin A on D7 following selection. Purity of primary γδ T cells was determined by flow cytometry and confirmed to be > 95% prior to downstream use.

*Co-culture experiments*

CAR-T cells were co-cultured in a black walled, clear bottomed 96-well plate, at a variety of effector: target ratios, with target cells retrovirally transduced then single-cell cloned to stably express Firefly luciferase with HA marker gene. 25000 target cells/ well were plated in RPMI with 10% FCS, in a total volume of 200uL/well. The plate was placed in a standard cell culture incubator containing 5% CO2 at 37 °C. After 48hrs the plate was centrifuged at 800G for 3mins and supernatant collected for analysis of cytokines, performed using Biolegend Human cytokine bead array kit (Biolegend, San Diego, USA). Residual tumour cells were then quantified by bioluminescence, recorded after addition of luciferin lysis buffer (*Bright Glo*, Promega, Wisconsin, USA). % cytotoxicity was recorded as (target signal – background signal)/ (targets alone signal – background signal)*100 ^2^.

For cytotoxicity experiments using primary γδ T cells as targets, autologous γδ T cells were incubated with effector cells as indicated, at a variety of effector: target ratios, with 50 000 target cells/ well at the start of the culture. After 5 days, the plate was spun down and supernatant removed. 100 μl of staining cocktail (with appropriate antibodies and viability dye (eBioscience, Thermo Fisher, Waltham, MA) diluted in PBS) was added, and cells were stained for 20 min in the dark at room temperature. Wells were then washed with an additional 100 μl of PBS and spun down at 800g for 3 min. Supernatant was decanted. Counting beads (Flow-Check Fluorospheres, BD Biosciences, NJ) were washed in PBS and resuspended at 5 × 10^4^ beads/ml in PBS. 100 μl of PBS/counting bead mixture was added to each well (500 beads/well). Approximately 200 beads were acquired per sample. Gating on single live target cells was performed according to exclusion of fixable viability dye, forward and side scatter characteristics, and expression of marker gene or fluorescent dye. Assays were performed in triplicate. Remaining cell numbers were calculated as 500/ number of beads collected × number of cells at end of culture.

*Murine experiments*

This work was performed under a UK Home Office–approved project license and was approved by the University College London Biological Services Ethical Review Committee. 6- to 8-week old male NSG mice (Jackson Laboratory, Bar Harbor, ME) were intravenously injected via the tail vein with Loucy cells, or CAR-T cells as described in the text. Tail vein bleeds of 50 μl were undertaken as indicated in the text. At the time of killing, bone marrow was collected in some experiments. Single-cell suspensions were prepared and analysed for the presence of T cells and residual Loucy cells by flow cytometry. Loucy cells were identified by expression of the HA marker gene. CAR T cells were identified by expression of the RQR8 marker gene. Gating strategy is shown in Supplementary Figure 1. For experiments with a survival endpoint, mice were weighed at least twice weekly. Animals with >10% weight loss or those displaying evidence of graft-versus-host disease or disease progression, including hunched posture, poor coat condition, reduced mobility, piloerection or hind limb paralysis, were killed.

Bioluminescence imaging of mice was performed using the IVIS system (Perkin Elmer, Buckinghamshire, UK). Prior to imaging, mice were placed in an anesthetic chamber. General anesthesia was induced using inhaled isoflurane. Following induction, intraperitoneal injection of luciferin (200 μl via 27-gauge needle) was undertaken. After 2 min, mice were placed in the imaging chamber. Simultaneous optical and bioluminescence imaging was performed. Anesthesia was maintained by continued inhalation of isoflurane during imaging.

*Statistical analyses*

Unless otherwise noted, data are summarized as mean ± sd. Student’s t-test was used to determine statistically significant differences between samples for normally distributed variables, and the Mann–Whitney U-test was used for nonparametrically distributed variables. P < 0.05 (two-tailed) indicated a significant difference. Unless otherwise stated, variances were similar between study populations. When variances were unequal, Welch’s correction for unequal variance was used. Paired analyses were used when appropriate. When multiple t-tests were performed, statistical significance was determined using the Holm–Sidak method with α = 0.05. For *in vivo* studies, mice were matched based on the luminescent signal for control and treatment groups before infusion of CAR-T cells. Investigators were blinded to the treatment allocation of mice. Cohort sizes were based on the number required to demonstrate 90% reduction in bioluminescence, 95% confidence with 80% power. Survival curves were generated using the Kaplan–Meier method, with hazard ratios calculated by the Mantel-Haenszel method. All mouse studies were performed at least twice. Graph generation and statistical analyses were performed using Prism version 8.4.2 software (GraphPad, La Jolla, CA).

*Data availability*

Data are available from the corresponding author upon request.

*Supplementary References*

1. Philip, B. *et al.* A highly compact epitope-based marker/suicide gene for easier and safer T-cell therapy. *Blood* **124**, 1277–1287 (2014).

2. Eyquem, J. *et al.* Targeting a CAR to the TRAC locus with CRISPR/Cas9 enhances tumour rejection. *Nature* **543**, 113–117 (2017).
