# Supplementary figures and images for: Chimeric antigen receptor T cells for gamma–delta T cell malignancies
Source: Leukemia. 2021 Aug 13;36(2):577–9. doi: 10.1038/s41375-021-01385-0 (PMC8807386; doi:10.1038/s41375-021-01385-0)

Supplementary  
Figure 1

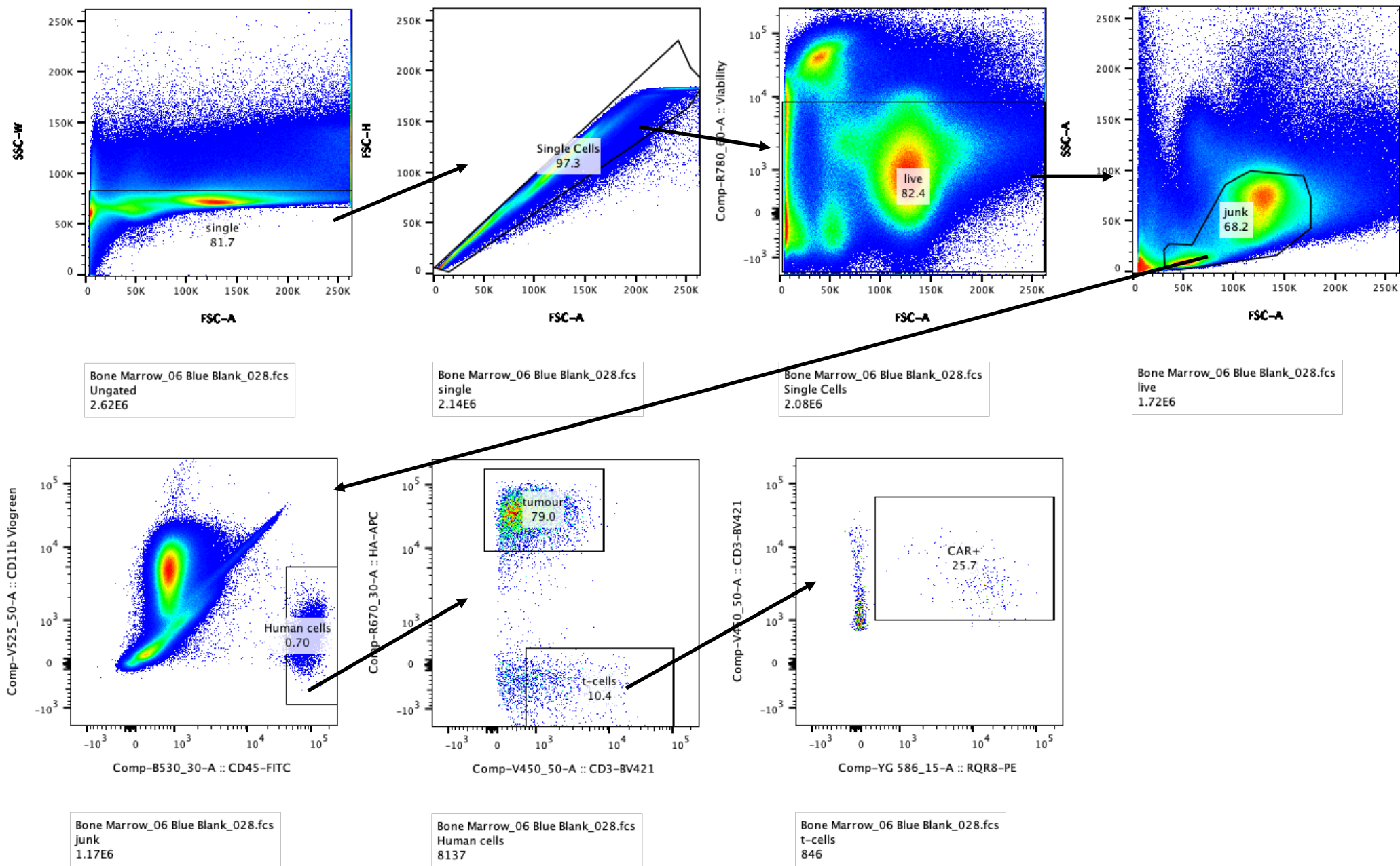

Supplement: Supplementary file 2 — Supplementary Figure 1 [file 41375_2021_1385_MOESM2_ESM.pdf]
